# Physicochemical properties of SARS‐CoV‐2 for drug targeting, virus inactivation and attenuation, vaccine formulation and quality control

**DOI:** 10.1002/elps.202000121

**Published:** 2020-06-08

**Authors:** Christin Scheller, Finja Krebs, Robert Minkner, Isabel Astner, Maria Gil‐Moles, Hermann Wätzig

**Affiliations:** ^1^ Institute of Medicinal and Pharmaceutical Chemistry Technische Universität Braunschweig Braunschweig Germany

**Keywords:** COVID‐19, Formulation, Physicochemical properties, SARS‐CoV, Vaccine

## Abstract

The material properties of the severe acute respiratory syndrome coronavirus 2 (SARS‐CoV‐2) and its proteins are discussed. We review the viral structure, size, rigidity, lipophilicity, isoelectric point, buoyant density and centrifugation conditions, stability against pH, temperature, UV light, gamma radiation, and susceptibility to various chemical agents including solvents and detergents. Possible inactivation, downstream, and formulation conditions are given including suitable buffers and some first ideas for quality‐control methods. This information supports vaccine development and discussion with competent authorities during vaccine approval and is certainly related to drug‐targeting strategies and hygienics. Several instructive tables are given, including the p*I* and grand average of hydropathicity (GRAVY) of SARS‐CoV‐1 and ‐2 proteins in comparison. SARS‐CoV‐1 and SARS‐CoV‐2 are similar in many regards, so information can often be derived. Both are unusually stable, but sensitive at their lipophilic membranes. However, since seemingly small differences can have strong effects, for example, on immunologically relevant epitope settings, unevaluated knowledge transfer from SARS‐CoV‐1 to SARS‐CoV‐2 cannot be advised. Published knowledge regarding downstream processes, formulations and quality assuring methods is, as yet, limited. However, standard approaches employed for other viruses and vaccines seem to be feasible including virus inactivation, centrifugation conditions, and the use of adjuvants.

**Abbreviations**
1‐PRF: programmed‐1 ribosomal frameshifting
COVID‐19: Coronavirus Disease 2019
CpG: cytosine‐phosphate‐guanosine
Cryo‐EM: cryoelectron microscopy
FCS: fetal calf serum
GRAVY: grand average of hydropathy
hACE2: human angiotensin‐converting enzyme 2
IEP: isoelectric point
MEM: minimum essential media
MERS: Middle East respiratory syndrome
NendoU: Nidoviral RNAuridylate‐specific endoribonuclease
Nsp: nonstructural protein
PDB: Protein Data Bank; http://www.wwpdb.org/
RBD: receptor‐binding domain
RBD‐S: receptor‐binding domain of the spike protein
RdRp: RNA‐dependent RNA polymerase
S/D: solvent/detergent
S^A^
: spike protein domain A
SARS‐CoV‐1: severe acute respiratory syndrome coronavirus 1
SARS‐CoV‐2: severe acute respiratory syndrome coronavirus 2
S^B^
: spike protein domain B
TCID_50_
: tissue culture infective dose 50
TNBP: tri(nbutyl) phosphate
VLP: virus‐like particle

## 1 Introduction

Knowledge about the physicochemical properties of the severe acute respiratory syndrome coronavirus 2 (SARS‐CoV‐2) is urgently needed to quickly develop live attenuated and inactivated vaccines. It is also important to understand these properties for related viral proteins, in order to develop subunit vaccines. Here, the spike protein of SARS‐CoV‐2 is certainly of major relevance, but others may also become of interest. Although several companies have already started to develop the first formulations for phase I clinical studies, this article should serve as a cross‐check for their own surveys [1]. This work may also be useful for discussing individual approaches with the competent authorities during vaccine approval. Knowledge about physicochemical properties is essential for developing suitable quality‐control methods for vaccines, but also for properly defining drug‐targeting strategies for small‐molecule pharmaceuticals. Furthermore, knowledge about SARS‐CoV‐2 inactivation certainly touches on the topics of hygienics and healthcare in general.

General information about SARS‐CoV‐2 and the Coronavirus Disease 2019 (COVID‐19) can be readily found in Wikipedia and on the internet. However, detailed information about physicochemical parameters is only available in the scientific literature. In particular, we considered:

- molecular weight;
- buoyant density and centrifugation conditions;
- the p*I* and pH stability;
- possible formulations, including suitable buffers;
- structure, rigidity, order and thermal stability; and
- lipophilicity and susceptibility to various physical and chemical agents, especially solvents and detergents.

The databases SciFinder and Google Scholar have been the main resources for this study, using the above‐mentioned keywords. In many cases, this information is not yet available or proprietary. Therefore, we also tried to derive information from articles about the severe acute respiratory syndrome coronavirus 1 (SARS‐CoV‐1), which caused the SARS pandemic in 2002 and the following years. If we did not find anything here either, we decided to have a look into publications about somehow related viruses, for example, (beta)coronaviruses in general. Comprehensive information about the taxonomy and similarities to other viruses is available [2, 3, 4].

We are conscious that deriving similar physicochemical properties from a faint taxonomical relationship is limited, since we learned that the immunogenic properties of SARS‐CoV‐2 and SARS‐CoV‐1 differ considerably [5]. Nevertheless, it is a place to start looking and one can derive approaches and procedures for investigating these parameters oneself using the information provided in these works.

As the need for research results on the present topic is high, working groups are striving to make results available as quickly as possible, and some materials are available online before they are accepted by a journal. It should therefore be noted that some sources cited in this article are preprints, that is, previously published online material, that is not yet peer‐reviewed [5, 6, 7, 8, 9, 10, 11, 12, 13, 14, 15, 16].

## 2 Size and structure

SARS‐CoV‐2 is categorized as a betacoronavirus. Its shape is round or elliptical and often pleomorphic, with the diameter varying between approximately 60 to 140 nm [17, 18]. SARS‐CoV‐2 is an enveloped virus (these have been nicely reviewed in [19]). The shapes of enveloped viruses differ considerably from one individual virus to another, since their lipophilic envelope can integrate varying amounts and types of proteins, allowing for a lot of flexibility. For SARS‐CoV‐1, size and shape differences are caused by different conformations of the M protein [20].

The single‐stranded RNA genome contains 29 891 nucleotides, encoding 9860 amino acids [17]. Besides the envelope (E) protein, three other structural proteins exist in SARS‐CoV‐2, as in the other *Coronaviridae* [19, 21]: S (spike protein), M (membrane protein), and N (nucleocapsid protein). Wu et al. [21] provide an excellent schematic illustration of the virus structure including the structural proteins. Details about the spike protein homotrimer, its subunits, and domains are competently given and illustrated in [22].

Zhu et al. present electron micrographs of SARS‐CoV‐2 particles which look generally spherical, but also show some pleomorphism. Distinctive spikes, about 9 to 12 nm long, protrude from the virus particle's surface, resembling a solar corona. This morphology can be found within the *Coronaviridae* family. Furthermore, it is described that free virus particles are found in the extracellular space and in membrane‐bound vesicle inclusion bodies filled with virus particles, which can be found in cytoplasm in the human airway epithelial ultrathin sections [18].

For comparison of the two SARS‐related coronaviruses known to date, a very detailed structural analysis of SARS‐CoV‐1 is provided in [23]. The genome of SARS‐CoV‐2 was reported recently; a high‐resolution map of the SARS‐CoV‐2 transcriptome and epitranscriptome has been presented using two complementary sequencing techniques [24].

All coronaviruses express E protein, a protein in the virus envelope with a transmembrane domain. It is not necessary for virus replication, but without it, the virus would be attenuated. The SARS‐CoV‐1 E protein was investigated regarding its structure, ion‐channel properties and why the drug hexamethylene amiloride, but not amiloride, has an inhibiting effect [25].

The interaction between the viral S protein and a host receptor has also been investigated by bioinformatics methods. The aim of the study was to find antibodies that can bind to SARS‐CoV‐2 S protein and have a neutralizing effect by interfering with the interaction between the S protein and the host receptor. Binding candidates, as potential lead substances, have been discussed [5].

It is known that SARS‐CoV‐1 recognizes human angiotensin‐converting enzyme 2 (hACE2) and uses it as entry receptor using the spike protein domain B (S^B^). The SARS‐CoV‐2 S protein is approximately 75% identical in its overall amino acid sequence to its CoV‐1 relative. Within the virus’ receptor binding domain (RBD), both S proteins are 50% identical [22, 26].

SARS‐CoV‐2 also uses hACE2 as an entry receptor to which it binds with similar affinity as SARS‐CoV‐1. Walls et al. [22] give a detailed summary of the entry mechanism, S protein subunits and distinct conformational changes. The SARS‐CoV‐2 S glycoprotein is formed by a transmembranal protein trimer that can adopt multiple, distinct conformational states. It comprises two functional subunits: S_1_ and S_2_. The S_1_ subunit is involved in binding to the host cell receptor. The S_2_ subunit is involved in the fusion of the viral and host cell membranes. Between both subunits, there is a protease cleavage site (S_2_’). Within the S_1_ subunit, distinct domains can be found in the different coronaviruses: domain A (S^A^) and domain B (S^B^); their function varies between the *Coronaviridae*. SARS‐CoV‐2 S^B^ and SARS‐CoV‐1 S^B^ also bind with comparable affinity to hACE2. Note that a cleavage site for the host protease furin has been identified at the SARS‐CoV‐2 S protein only. This is cleaved during biosynthesis. The site is formed by insertion of four additional amino acids. The virus entry mechanism therefore mainly consists of four steps. First, the complete S protein binds to the ACE2 receptor. Second, furin cleavage takes place and forms S_1_ and S_2_. This opens the S protein structure for the third step, another protease cleavage. Consequently, the conformational changes of the bound S protein at the receptor after cleavage facilitate the virus entry by orders of magnitude [27]. Altering the cleavage sites could affect virus entry, and consequently, alter pathogenicity and transmissibility.

Cryo‐electron microscopy (cryo‐EM) structures of the SARS‐CoV‐2 S protein ectodomain trimer are presented in [22]. The S^B^ domain is found in multiple conformations, as can be expected from similar findings for SARS‐CoV S and Middle East respiratory syndrome coronavirus (MERS‐CoV) S proteins. The authors tested antiserum containing murine polyclonal antibodies against SARS‐CoV‐1 S protein. Virus entry into target cells could be inhibited [22].

Yan et al. describe the structural basis for recognition of SARS‐CoV‐2 by hACE2, which acts as a receptor for the RBD of the surface S glycoprotein. The cryo‐EM structure of the full‐length hACE2 bound to the RBD of SARS‐CoV‐2 is presented with a resolution of 3.5 Å at the interface. Details of the overall structure of the complex and the interface are given. Furthermore, the authors have compared differences and similarities between the respective interfaces of SARS‐CoV‐1 and SARS‐CoV‐2 in complex with hACE2 [16, 28].

The crystal structure of SARS‐CoV‐2 RBD bound with ACE2 at 2.45 Å resolution is presented by Lan et al. The SARS‐CoV‐2 RBD (residues Arg319‐Phe541) and the N‐terminal peptidase domain of ACE2 (residues Ser19‐Asp615) were cocrystallized. The complex structure was solved by molecular replacement using SARS‐CoV‐1 RBD and ACE2 structures as search models. The complex structure is described in detail. Similarities and differences between SARS‐CoV‐2 RBD/ACE2 and SARS‐CoV‐1/ACE2 are discussed [15].

The neutralizing antibody CR3022 was previously isolated from a convalescent SARS patient. A cross‐reactive binding to SARS‐CoV‐1, but also to SARS‐CoV‐2, was postulated. Therefore, the crystal structure of the RBD of the SARS‐CoV‐2 S protein in complex with CR3022 has been determined and described in detail (Protein Data Bank (PDB) ID: 6W41) [4]. Although the binding epitope is highly conserved when comparing SARS‐CoV‐1 and SARS‐CoV‐2, the binding affinity to the SARS‐CoV‐1 epitope is higher. The authors discuss this difference and also include structural information known from cryo‐EM structures given by Wrapp et al. [29] and by Walls et al. [22]. Yuan et al. further describe their results from examining the hinge‐like movement of the S protein trimer's RBD on binding possibilities for CR3022. The RBD monomer can move between an “up” and a “down” conformation, some up/down combinations of the RBD trimer can conflict with the CR3022 antibody and interfere with its binding. The authors discuss their results of structural modeling investigating the effect of different combinations of the “up” and “down” conformations on the accessibility of the binding site for CR3022 [4].

There is also a brief, but noteworthy, communication regarding the SARS‐CoV‐1 S protein with good cryo‐EM images for comparison [30], and a study about the SARS‐CoV‐1 S protein and the stability needed for the fusion protein to merge with the host cell, whereby hydrophobic residues play a major role in the post‐fusion stability [31].

Despite some structural differences, in serological studies, it might be difficult to distinguish exposures to SARS‐CoV‐2 from other related SARS‐CoV viruses, in so far as the assay is based on S ectodomain trimers. More specific assays are probably required [22].

Nevertheless, distinctly different immunogenic properties related to SARS‐CoV‐1 might be expected. The antigenic, structural and glycosylation differences from the S proteins of SARS‐CoV‐1 or ‐2 have been compared [32].

SARS‐CoV‐2 and SARS‐CoV‐1 show an amino acid sequence homology of 75.5% within the RBD. However, comparing the S glycoproteins in SARS‐CoV‐2 and SARS‐CoV‐1, numerous novel antigenic epitopes have been found. Considering the sequence differences of approximately 25%, a related share of novel epitopes in the RBD of SARS‐CoV‐2 S protein would be expected, but the new ones contributed to 85.3% of all of the antibody epitopes, demonstrating that the morphological and immunological similarities are much lower than the sequence homology [33].

D'Annessa et al. performed a comparative analysis of already and recently published protein structures of SARS‐CoV‐2 and homologous viruses. Their aim was to find common and distinctive traits on the protein surface that underlie the recognition mechanisms of cell receptors and the immune‐system molecules with their recently developed prediction method, Matrix of Low Coupling Energies (MLCE). This method was applied to predict subsets of surface residues that could be antibody‐binding epitopes and protein‐protein interaction regions of the RBD of S proteins from SARS‐CoV‐2 and SARS‐CoV‐1 [10].

Other possibly interesting protein structures include the M protein, the SARS protease, the nidoviral RNAuridylate‐specific endoribonuclease (NendoU), corresponding to Nsp15 (nonstructural protein 15) and the RNA‐dependent RNA polymerase (RdRp). Zhu et al. investigated the protease and presented four crystal structures of SARS‐CoV‐1 main protease (M^pro^) (PDB ID: 2H2Z) in complex with pentapeptide aldehydes (Ac‐ESTLQ‐H, Ac‐NSFSQ‐H, Ac‐DSFDQ‐H, and Ac‐NSTSQ‐H) [34], as well as the N protein and Nsp2 (see section 8 “Electrophoresis”).

A follow‐up investigation of one group focused on the structural investigations of the SARS‐CoV‐1 M protein. They investigated viruses and virus‐like particles (VLPs) via different constructs of the M protein using cryo‐EM, tomography, and statistical analysis. The M protein of coronaviruses plays a central role in virus assembly regarding the combination of virus and host factors to make new virus particles. The M protein is a functional dimer. Two conformations M_LONG_ and M_COMPACT_ have been described. The elongated form is associated with rigidity, clusters of spikes, and a relatively narrow range of membrane curvature, while the compact M protein is associated with flexibility and low spike density. Differences in particle size and the efficiency of virus assembly due to interactions of the M protein with other proteins have been investigated with a model [20].

It was shown that the SARS‐CoV‐1 3a protein is a membrane‐associated protein. It is colocalized and interacting with the M protein. This indicates that 3a could be related to the virus budding. As a membrane‐associated protein, it may be a target for immune‐system recognition and vaccines [35]. Another publication also reported the membrane association of the SARS‐CoV‐1 3a protein. Moreover, they mentioned some evidence that 3a proteins may have a subpopulation, which is associated with membranes resistant to treatment with detergents [36].

Two crystal structures of a protease from SARS‐CoV‐2 were recently reported: free (PDB ID: 6Y2E) and inhibitor bound protease (PDB ID: 6LU7). The authors produced flexible variations on the crystal structures by computational investigation methods to generate a database. These generated PDB files have been made available in a database to facilitate further investigations, for example, on new binding sites [13].

The crystal structure of Nsp15 endonuclease NendoU from SARS‐CoV‐2 has been presented with 2.20‐45.10 Å resolution (PDB ID: 6vww). The Nsp15 consists of a 39 kDa monomeric unit which folds into three domains. The active protein forms hexamers made from dimers of trimers. A channel runs through this hexamer, which is essential for enzymatic activity. Nsp15 has Mn^2+^ dependent endonuclease activity that cuts dsRNA. Active sites are located on top and bottom of the assembly and all six binding sites can possibly be occupied simultaneously, though no structure of the protein/RNA‐complex is yet available.

The structural comparison suggests that inhibitors of SARS‐CoV‐1 Nsp15 have a good chance of inhibiting the SARS‐CoV‐2 Nsp15 as well, but perhaps not MERS‐CoV NendoU. Furthermore, a crystal structure of the Nsp15 citrate complex at 1.9 Å resolution will be made available under PDB ID: 6wo1 [37].

Another important virus protein is the nucleocapsid protein N, which is the main protein of the virus capsid. It is not accessible to vaccines. Studies about the stability of the SARS‐CoV‐1 nucleocapsid protein N [38] and its electrostatic interactions with the SARS‐CoV‐1 membrane protein [39] have also been reported.

Physicochemical information about the 3‐chymotrypsin‐like cysteine protease (3CL^pro^) enzyme from SARS‐CoV‐2 have also already been investigated, as this protein controls the coronavirus replication and is essential for its life cycle [40].

The known antiviral drugs Sofosbuvir, Alovudine, and Zidovudin were examined as inhibitors of SARS‐CoV RdRp using model polymerase extension experiments. The activated triphosphate forms of Sofosbuvir, Alovudine, and Zidovudin were incorporated by SARS‐CoV RdRp and terminated further polymerase extensions. Due to a 98% amino acid similarity of the SARS‐CoV‐1 and SARS‐CoV‐2 RdRps, the authors expect these nucleotide analogues to inhibit the SARS‐CoV‐2 polymerase, and when further modified, they could generate more potent drug candidates [11].

In search of a potential drug target for small molecules, Kelly and Dinman examined a molecular mechanism called programmed‐1 ribosomal frameshifting (‐1 PRF). It controls the relative expression of the proteins in all coronaviruses. Insertion of point mutations lowers ‐1 PRF activity and virus replication [14].

## 3 Isoelectric point and pH stability

The isoelectric point (abbreviated by p*I* or IEP) is the pH value at which a molecule's or biocolloid's net charge is zero. The latter includes viruses, which can form different surface charges, depending on the pH [41]. Information about the isoelectric point is important because the solubility and electrical repulsion are lowest at the p*I*. Hence, the tendency to aggregation and precipitation is highest [42]. In the case of viruses, the value thus provides information about the viral surface charge in a specific environment [41, 43]. Michen and Graule authored a review on isoelectric points of viruses in which they evaluated 137 p*I* measurements of 104 viruses [41]. Their evaluation shows that the isoelectric points (IEP) of viruses range from 1.9 to 8.4, but can mostly be found between 3.5 and 7, which means that viruses with p*I* values in the strongly basic range have not yet been described. They offer various attempts to explain the variations found in single‐virus species data. Key propositions are that viruses can have more than one p*I* and that the p*I* value depends on the electrolyte conditions to which they are exposed. Dependencies of charge states, and thus, p*I* values, on the presence of metal ions (for example) have been frequently observed [44, 45, 46]. At present, no experimental data are available for the p*I* value of SARS‐CoV‐2 or its structural, nonstructural, or accessory proteins. However, an estimation is possible through predictive calculations with, for example, the ProtParam tool by ExPASy [47]. The calculation results for the physicochemical properties and the molecular weight of the individual proteins are based on the corresponding amino acid sequences from UniProt [48] and are shown in Table 1. Please note that these tools do not take post‐translational modification into account; therefore, these estimations need to be read with caution.

**Table 1:** Molecular Weight (MW), isoelectric Point (p*I*), and grand average of hydropathicity (GRAVY) of the SARS‐CoV‐1 and SARS‐CoV‐2 proteins predicted by ProtParam [54] including the corresponding sequence identifier [48]

Virus	Protein	UniProt‐ID	MW [kDa]	p*I*	GRAVY
SARS‐CoV‐1	Replicase polyprotein 1a	P0C6U8 · R1A_CVHSA	486.373	5.91	–0.020
	Replicase polyprotein 1ab	P0C6 × 7 · R1AB_CVHSA	790.248	6.19	–0.071
	Spike glycoprotein	P59594 · SPIKE_CVHSA	139.125	5.56	–0.045
	Nucleoprotein	P59595 · NCAP_CVHSA	46.025	10.11	–1.027
	Protein 3a	P59632 · AP3A_CVHSA	30.903	5.75	0.239
	Protein 7a	P59635 · NS7A_CVHSA	13.941	8.24	0.218
	Envelope small membrane protein	P59637 · VEMP_CVHSA	8.361	6.01	1.141
	Membrane protein	P59596 · VME1_CVHSA	25.061	9.63	0.417
	Nonstructural protein 3b	P59633 · NS3B_CVHSA	17.750	10.82	0.099
	Nonstructural protein 6	P59634 · NS6_CVHSA	7.527	4.64	0.297
	Protein 9b	P59636 · ORF9B_CVHSA	10.802	4.90	–0.122
	Protein nonstructural 7b	Q7TFA1 · NS7B_CVHSA	5.302	3.77	1.414
	Nonstructural protein 8b	Q80H93 · NS8B_CVHSA	9.560	9.45	–0.029
	Nonstructural protein 8a	Q7TFA0 · NS8A_CVHSA	4.327	8.30	0.644
	Uncharacterized protein 14	Q7TLC7 · Y14_CVHSA	7.852	6.25	0.310
SARS‐CoV‐2	Replicase polyprotein 1a	P0DTC1 · R1A_SARS2	489.989	6.04	–0.023
	Spike glycoprotein	P0DTC2 · SPIKE_SARS2	141.178	6.24	–0.079
	Replicase polyprotein 1ab	P0DTD1 · R1AB_SARS2	794.058	6.32	–0.070
	Protein 3a	P0DTC3 · AP3A_SARS2	31.123	5.55	0.275
	Membrane protein	P0DTC5 · VME1_SARS2	25.147	9.51	0.446
	Protein 7a	P0DTC7 · NS7A_SARS2	13.744	8.23	0.318
	Nucleoprotein	P0DTC9 · NCAP_SARS2	45.626	10.07	–0.971
	Envelope small membrane protein	P0DTC4 · VEMP_SARS2	8.365	8.57	1.128
	Nonstructural protein 6	P0DTC6 · NS6_SARS2	7.273	4.60	0.233
	Protein 9b	P0DTD2 · ORF9B_SARS2	10.797	6.56	–0.085
	Nonstructural protein 8	P0DTC8 · NS8_SARS2	13.831	5.42	0.219
	Uncharacterized protein 14	P0DTD3 · Y14_SARS2	8.050	5.79	0.603
	Protein nonstructural 7b	P0DTD8 · NS7B_SARS2	5.180	4.17	1.449
	A0A663DJA2 · A0A663DJA2_SARS2		4.449	7.93	0.637

The structural proteins S, E, and M might be of major importance for the development of possible vaccines because they play a key role in the entry of the virus into the host cell or in the assembly of the virus [8, 9]. The p*I* values of S, E, and M are 6.24, 8.57, and 9.51, respectively. These calculated values agree with the calculations from other research groups [7, 8, 9]. In addition to ProtParam, there is another freely accessible tool on the Internet for calculating p*I* values based on amino acid sequences, Proteome‐*pI*. Proteome‐*pI* is a database with predicted p*I* values for over 5000 proteomes [49]. It also offers the possibility of predicting p*I* values of novel amino acid sequences using 18 different methods. In the context of predictive tools, it is worth mentioning that the performance of these depends on the quality of the underlying database. The obtained results certainly depend on the modelled environment, and hence, also on the concentration of various metal ions [44, 45, 46].

There is a review article on initial successes in the identification and management of coronavirus disease in 2019 [50]. It contains a section on the physicochemical properties of SARS‐CoV‐2. Among others, the IEP, the instability index, and the grand average of hydropathicity (GRAVY) are predicted. Reference is made to the SARS‐CoV‐2 polypeptide by specifying the sequence ID of the genome (MN908947.3). However, it does not become completely clear what the term “SARS‐CoV‐2 polypeptide” means. The resulting values suggest that this is the genome translated into one continuous amino acid sequence. However, it has to be considered that such a polypeptide will not be existent in this form. This could also be the reason why the instability index given there indicates that the protein is unstable. In view of these assumptions, the extent to which this information is helpful is questionable.

The pH is a key factor to take into account because its changes can produce significant alterations in the structure of proteins. A change in the conformation of viral proteins that are involved in attachment to, and replication in, a host cell can even lead to inactivated viruses.

As mentioned above, a pH change in the direction of the IEP may cause insolubility or precipitation. The p*I* and pH stability parameters can therefore be related to each other. Chin et al. suitably examined the stability of SARS‐CoV‐2 in different environmental conditions including different temperatures, surfaces, disinfectants, and different pH values [51]. Regarding pH, they found that the virus is extremely stable over a wide pH range, namely pH 3‐10.

Darnell et al. [52] carried out a study to evaluate the effect of pH on the infectivity of SARS‐CoV‐1 since the pH can affect the conformation of the S protein of the virus [52, 53]. To evaluate the effect of the pH, virus aliquots were adjusted to the desired pH using 5 M and 1 M HCl or 5 M and 1 M NaOH. Subsequently, they were divided into three aliquots, incubated at different temperatures (4, 25, and 37°C), neutralized to pH 7, and analyzed for viral titer.

It was observed that exposure of SARS‐CoV‐1 for 1 h to extreme alkaline pH (12 and 14) led to complete inactivation of the virus, regardless of temperature (4, 25, and 37°C). Moderate pH values from 5 to 9 had only little effect on the virus titer at all testing temperatures. On the other hand, at very acidic pH conditions (pH 1 and 3), the virus is completely inactivated at 25°C and 37°C. By contrast, at 4°C, a decrease in infectivity is observed, although the virus is not completely inactivated. In conclusion, the infectivity of SARS‐CoV‐1 is sensitive to pH extremes.

## 4 Lipophilicity

The susceptibility of viruses to disinfectants depends on whether they are lipophilic or hydrophilic in nature, that is, whether they have a lipid envelope or not [55]. In 1983, Klein and Deforest classified viruses into three groups: A (lipophilic with envelope), B (hydrophilic, nonenveloped), and C (intermediate solubility, nonenveloped). Furthermore, they divided disinfectants into two groups: the lipophilic agents that failed to inactivate nonlipid viruses and the broad‐spectrum agents inactivating all viruses. Table 2 shows the classification of viruses and disinfectants according to Klein and Deforest [56].

**Table 2:** Klein and Deforest classification of viruses and disinfectants [55]

			Sensitivity to disinfectants	
Viral group	Lipid envelope	Examples of viruses	Lipophilic	Broad spectrum
A	✓	Herpes simplex virus, human immunodeficiency virus, influenza virus, coronaviruses	✓	✓
B	✗	Nonlipid picornaviruses, parvoviruses	✗	✓
C	✗	Adenovirus, reovirus	✗	✓

Since coronaviruses, and therefore SARS‐CoV‐2, have an envelope, they should be in group A, according to the Klein and Deforest classification system, as shown in Table 2. Thus, SARS‐CoV‐2 might allow the penetration of lipophilic antimicrobials such as halogens, aldehydes, quaternary ammonium compounds (QAC), phenolics, alcohols, peroxides, proteases, and detergents [57]. Srivastava et al. examined the effectiveness of various malaria drugs against SARS‐CoV‐2 and found that the more lipophilic the drug, the better it can penetrate the virus and inhibit it. Possible active substances against SARS‐CoV‐2 should therefore be of a lipophilic nature in order to penetrate the viral membrane and inactivate the virus [6]. However, it must be noted that the penetration of a substance into the virus is not always directly associated with the loss of the replication functionality of the nucleic acid and its complete demolition [58]. The infection of host cells by enveloped viruses is based on the fusion of the virus envelope with the endosomal or plasma membrane of the cell. The protein and lipid compositions of the virus envelope, as well as the host cell membrane, thus, play a decisive role in enveloped virus infection [59]. Using the ProtParam tool from the Bioinformatics Resource Portal ExPASy [54], the grand average of hydropathicity index (GRAVY) of the individual proteins of SARS‐CoV‐1 and SARS‐CoV‐2 was calculated using the amino acid sequences found on the Universal Protein Resource (UniProt) [48]. Table 1 shows that the individual proteins of the two viruses hardly differ in their hydrophobicity.

## 5 Virus surface stability and inactivation

To reduce the spread of SARS‐CoV‐2, it is indispensable to know the stability of the virus under several conditions and the substances that are able to inactivate it and make it harmless. Therefore, as reported in their short letter [59], van Doremalen et al. investigated the surface stability of SARS‐CoV‐1 and SARS‐CoV‐2 in five environmental conditions, namely aerosols, plastic, stainless steel, copper, and cardboard. Both SARS‐CoV viruses remained viable during the whole aerosol experiment (3 h), with a similar reduction of virus concentration (10^3.5^ to 10^2.7^ tissue culture infective dose 50 (TCID_50_) for SARS‐CoV‐2 versus 10^4.3^ to 10^3.5^ TCID_50_ for SARS‐CoV‐1). SARS‐CoV‐2 was stable on plastic and stainless steel for the longest time, whereas viable virus was detectable up to 72 h. However, both viruses had an exponential decay on all investigated conditions and similar estimated half‐lives, except for the cardboard. For the latter, the “noise” of the individual experiments was also higher; therefore, this result has to be interpreted with caution. SARS‐CoV‐2 had the longest half‐lives on plastic and stainless steel, with 6.8 and 5.6 h, respectively [60].

Based on the previously mentioned publication, it can be assumed that SARS‐CoV‐2 has surface stabilities similar to those of SARS‐CoV‐1. A review showed, among other things, that depending on the conditions, SARS‐CoV‐1 persisted up to 9 days on plastic. However, recommended biocidal agents for disinfection are able to significantly decrease the viral infectivity within a short time [61]. Regarding disinfection; a similar trend to that in the previous study was shown by another study for SARS‐CoV‐1 [62].

### 5.1 Inactivation by heat and radiation

SARS‐CoV‐2 is relatively stable against heat and UV or gamma radiation. Several authors have evaluated the thermal inactivation of SARS‐CoV‐1 [52, 53, 63]. After treatment of SARS‐CoV‐1 at 56°C for different periods of time, it was observed that after 20 min of incubation, the infectivity was below the LOD. However, 60 min or more are necessary for complete inactivation. At 65°C, most of the viruses were inactivated if incubated for longer than 4 min and only 10 min were needed to bring the infectivity close to the LOD. However, again it takes 60 min or more for the virus to be completely inactivated. Instead, at 75°C, SARS‐CoV was completely inactivated in 45 min. In conclusion, thermic treatments can inactivate viruses, but some heat‐resistant particles may remain in the inactivated samples.

The ultraviolet light (UV) range is divided into UVA, UVB, and UVC. UVC (200‐280 nm) provides the most energetic UV light that can be absorbed by RNA or DNA. Darnell et al. [52] studied the effect of UVA and UVC. The virus stocks were placed in 24‐well tissue culture plates and exposed to UV irradiation (distance of 3 cm from the bottom of the wells). Exposure of the virus to UVA had no significant effect on SARS‐CoV infectivity even after 15 min of exposure. By contrast, UVC exposure produced a partial inactivation in 1 min, but it took 15 min for the virus to be completely inactivated. Subsequently, the same authors determined the effect produced by the presence of the BSA protein during the process of inactivating the virus with UVC radiation [63]. In this study, it was determined that the presence of BSA produced a protective effect. The virus was not inactivated even after 60 min of exposure, regardless of the BSA concentration being used (10, 16, or 25%).

Gamma radiation can inactivate biological material by the dislocation of electrons, the breakage of covalent bonds, or indirect damage via free radicals formed after the breakage of covalent bonds. A study evaluated the capacity of gamma radiation in the process of inactivation of SARS‐CoV‐1 [52]. In this study, a SARS‐CoV‐1 solution (400 µL of 10^6.33^ TCID_50_ per mL) was subjected to gamma radiation (30, 50, 100, and 150 Gy) from a ^60^Co source. However, no significant effect on virus infectivity was observed after 15 min of exposure.

### 5.2 Chemical agents

#### 5.2.1 Disinfectants

Rabenau et al. [62, 64] carried out studies to evaluate how different disinfectants affect SARS‐CoV‐1 (Tables 3 and 4). They studied 2‐propanol (70 and 100%), Desderman® N (78% ethanol, 0.2% 2‐biphenylol), Sterillium® (45% 2‐propanol, 30% 1‐propanol), formaldehyde (0.7% and 1%), glutardialdehyde (0.5%), Incidin™ plus (2%; containing 26% glucoprotamin), and in addition, wine vinegar (acid concentration 6%, sugar concentration 5% w/v) [62].

**Table 3:** Virus titer of SARS‐CoV‐1 with the corresponding minimal reduction factor (MRF) after treatment with various disinfectants (compiled from [62])

Treatment	Virus titer (TCID_50_/mL [log10])	MRF (log10)
100% 2‐Propanol	≤1.8 (30 s)	≥3.31
70% 2‐Propanol	≤1.8 (30 s)	≥3.31
Desderman®	≤1.8 (30 s)	≥5.01
Sterilium®	≤3.8 (30 s)	≥2.78
0.7% Formaldehyde	≤3.8 (120 s)	≥3.01
1% Formaldehyde	≤3.8 (120 s)	≥3.01
0.5% Glutaraldehyde	≤2.8 (120 s)	≥4.01
2% Incidin™ plus	≤4.8 (120 s)	≥4.01
Wine vinegar	≤2.8 (60 s)	≥3.00

**Table 4:** Minimal reduction factor (MRF) of SARS‐CoV‐1 after treatment with various disinfectants for several exposure times (compiled from [64])

		MRF (log10)		
Treatment	Exposure time	0.3% BSA	10% FCS	0.3% BSA + 0.3% sheep erythrocytes
Sterillium®	30 s	≥4.25 (0.47)	≥4.25 (0.47)	≥4.25 (0.47)
Sterillium® Rub	30 s	≥4.25 (0.47)	≥4.25 (0.47)	≥4.25 (0.47)
Sterillium® Gel	30 s	≥5.50 (0.54)	≥5.50 (0.54)	≥5.50 (0.54)
Virugard®	30 s	≥5.50 (0.54)	≥5.50 (0.54)	≥5.50 (0.54)
Mikrobac® forte	30 min	≥6.13 (0.35)	≥6.13 (0.35)	≥6.13 (0.35)
	60 min	≥6.13 (0.35)	≥6.13 (0.35)	≥6.13 (0.35)
Kohrsolin® FF	30 min	≥3.75 (0.71)	≥3.75 (0.71)	≥3.75 (0.71)
	60 min	≥3.75 (0.71)	≥3.75 (0.71)	≥3.75 (0.71)
Dismozon® pur	30 min	≥4.50 (0.54)	≥4.50 (0.54)	≥4.50 (0.54)
	60 min	≥4.50 (0.54)	≥4.50 (0.54)	≥4.50 (0.54)
Korsolex® basic	15 min	≥3.25 (0.47)	≥3.25 (0.47)	≥3.25 (0.47)
	30 min	≥3.25 (0.47)	≥3.25 (0.47)	≥3.25 (0.47)
	60 min	≥3.25 (0.47)	≥3.25 (0.47)	≥3.25 (0.47)

In another study, they evaluated four alcohol‐based hand disinfectants: Sterillium®, based on 45% isopropanol, 30% *n*‐propanol, and 0.2% mecetronium etilsulphate; Sterillium® Rub, based on 80% ethanol; Sterillium® Gel, based on 85% ethanol; and Sterillium® Virugard, based on 95% ethanol. Three additional products were surface disinfectants: Mikrobac® forte, based on benzalkonium chloride and laurylamine; Kohrsolin® FF, based on benzalkonium chloride, glutaraldehyde, and didecyldimonium chloride; and Dismozon® pur, based on magnesium monoperoxyphthalate, as well as the instrument disinfectant Korsolex® basic, based on glutaraldehyde and (ethylenedioxy)dimethanol. In this study, the authors also evaluated the presence of organic load (0.3% albumin, 10% fetal calf serum (FCS), or 0.3% albumin with 0.3% sheep erythrocytes).

To carry out these experiments, eight volume equivalents of the compound (adapted to room temperature) were mixed with one volume equivalent of the virus suspension and one volume equivalent of minimal essential medium (MEM) or organic load (0.3% albumin, 10% FCS, and 0.3% albumin with 0.3% sheep erythrocytes). After incubation at room temperature for different periods of time, the mixtures were put into an ice bath to avoid an extension of the effective incubation period. Subsequently, to evaluate the viral activity, the solutions were diluted 10 times with ice‐cold MEM. If the cytotoxic effect of a disinfectant was still present at a dilution of 1:1000, the virus‐disinfectant mixture was membrane filtered after incubation using Amicon® Ultra‐4 Filter units 100 kDa [64].

The solvents 2‐propanol (70 and 100%), Desderman® N (78% ethanol, 0.2% 2‐biphenylol), and Sterillium® (45% 2‐propanol, 30% 1‐propanol) were able to inactivate SARS‐CoV‐1 within 30 s of contact. In the case of formaldehyde (0.7% and 1%), glutardialdehyde (0.5%) and Incidin™ plus (2%; containing 26% glucoprotamin), the virus became noninfectious after 120 s of incubation. Finally, they also evaluated the effect of wine vinegar, obtaining a reduction factor ≥3log_10_ after 60 s (Table 3, [62]).

The results obtained indicate that all disinfectants are active against SARS‐CoV, regardless of the type of organic load.

Another study evaluated the ability of PVP‐iodine (PVP‐I) products to inactivate the SARS virus [53]. They tested the efficacy of several PVP‐I products including Isodine®, Isodine® Scrub, Isodine® Palm, Isodine® Gargle, and Isodine® Nodo Fresh. All of them are used as disinfectants for medical instruments and skin, as well as for hand washing, gargling, and spraying the throat. Aliquots of stock virus (0.1 mL) were mixed with an equal volume of various PVP‐I products. The mixtures were incubated for 1 min at room temperature and then diluted 10 times with sodium thiosulfate (0.5%) to neutralize the cytotoxicity and antiviral activity of PVP‐I. The mixtures were serially diluted in MEM and 0.1 mL aliquots.

Treatment of SARS‐CoV‐1 for 1 min with Isodine® Scrub, Isodine® Palm, and Isodine® Nodo Fresh reduced the virus infectivity below the LOD. By contrast, using Isodine® and Isodine® Gargle, it took 2 min to completely inactive the virus (Table 5).

**Table 5:** Virus titer of SARS‐CoV‐1 after treatment with various PVP‐I products for 1 or 2 min (compiled from [53])

	Virus titer (TCID_50_/mL)	
Treatment	1 min	2 min
Control	1.17 × 10^6^	<LOD
Isodine®	95.1	<LOD
Isodine® Gargle	190	<LOD
Isodine® Scrub	<LOD	Not done
Isodine® Palm	<LOD	Not done
Isodine® Nodo Fresh	<LOD	Not done

In addition, ether (75%), ethanol, chlorine‐containing disinfectant, peroxyacetic acid, and chloroform have been mentioned to effectively inactivate SARS‐CoV‐2 [17]. We only found this single reference mentioning the effect of ether and chloroform, thus, we have considered it important to mention. However, no data have been provided here, which underline the need for future investigations.

#### 5.2.2 Fixation solutions

The evaluation of the efficacy of different commonly used fixation procedures in eliminating the infectivity of SARS‐CoV viruses have been discussed in several articles.

Rabenau et al. [62] evaluated the efficacy of 100% acetone, acetone/methanol mixture (40:60), 100% ethanol, 70% ethanol, and a mixture of ethanol and phosphate‐buffered saline (1+1). To this end, SARS‐CoV‐1‐infected Vero cells were fixed for different periods of time using the various mixtures. After storage at ‐80°C for 24‐72 h, the cells were scratched from the slide, resuspended in MEM, and inoculated onto confluent Vero cells.

Most of the fixative solutions were capable of completely inactivating the virus. Cold acetone took 90 s to completely inactivate the virus. With ice‐cold acetone/methanol mixture (40:60), 10 min were needed; 70% ethanol took 10 min, and only 5 min were necessary using 100% ethanol. However, after fixation with a 1+1 mixture of phosphate‐buffered saline (PBS) and 100% ethanol, after 5 min, low‐level residual infectivity was observed, but was not quantified.

Kariwa et al. [53] evaluated the efficacy of 100% acetone, 100% methanol, 3.5% paraformaldehyde, and 2.5% glutaraldehyde for various times. SARS‐CoV‐1‐infected Vero cells were suspended in acetone and held at ‐10°C in a freezer. The cells suspended in the other reagents were held at room temperature. After this treatment, the cells were collected by centrifugation, washed with PBS, suspended in 1 mL MEM, and inoculated onto confluent Vero cells. After treatment with any of the fixatives, no infectivity remained in the cells (see Table 6).

**Table 6:** Virucidal activity of various fixation solutions against SARS‐CoV‐1 after several exposure times (compiled from [53])

	Virus titer (TCID_50_/mL)					
Treatment	0 min	5 min	15 min	30 min	60 min	90 min
Methanol	2.1 × 10^7^	Not done	Not done	<20	<20	<20
Acetone	1.3 × 10^7^	<20	<20	<20	<20	Not done
2.5% glutaraldehyde	2.2 × 10^6^	<160	<80	<80	<80	Not done
3.5% paraformaldehyde	1.6 × 10^6^	<320	<320	<320	<320	Not done

Therefore, in all cases, the different fixative solutions were capable of completely inactivating the virus, with differences in the necessary incubation times.

#### 5.2.3 Solvent/Detergent

Solvent/detergent (S/D) treatment is a standard method used to inactivate viruses in human blood products. The S/D treatment causes enveloped viruses to be irreversibly destroyed. Several methods are useful and should minimize the potential risk of transmission of viruses from components derived from human plasma. It is important to know how the use of different S/D treatments affects the SARS‐CoV viruses.

The S/D treatment of OCTAGAM (manufactured by Octapharma Pharmazeutika Produktionsges. m.b.H., Vienna, Austria) has proven to be effective in destroying enveloped viruses. To evaluate the efficacy on SARS‐CoV‐1, the industrial scale was adapted to the laboratory scale [65].

The industrial scale included treatment with 0.3% w/w tri(nbutyl) phosphate (TNBP) and 1.0% w/w Octoxynol (trade name: Triton X‐100) at 6.0 ± 0.5°C and pH 5.3 ± 0.2 for a minimum of 4 h. On the laboratory scale, lower concentrations of solvent and detergent (75% of standard S/D concentration) have been employed with a shortened processing time (30 min).

To determine the inactivation of SARS‐CoV‐1, infectivity was evaluated at different incubation times (1, 3, 5, 10, 20, and 30 min). The S/D treatment was terminated by a 1:250 dilution with a cell‐culture medium. The diluted test samples were screened with Vero cells (see Table 7). The results obtained reflect the high capacity of the OCTAGAM method to inactivate SARS‐CoV‐1, since the detection limit was reached within 1 min of S/D exposure.

**Table 7:** Virus titer after S/D treatment for several exposure times (compiled from [65])

Exposure time(min)	Virus titer (log_10_ TCID_50_/mL)	Reduction factor log_10_
1	≤1.37	≥4.56 ± 0.25
3	≤1.37	≥4.56 ± 0.25
5	≤1.37	≥4.56 ± 0.25
10	≤1.37	≥4.56 ± 0.25
20	≤1.37	≥4.56 ± 0.25
30	≤0.18	≥5.75 ± 0.25

In another article, the authors evaluated the efficacy of different S/D treatments (TNBP/Triton X‐100, TNBP/Tween 80, and TNBP/sodium cholate), as well as the influence of the use of different buffers: PBS or BSA‐PBS [63]. The virus was diluted in BSA‐PBS protein solution or PBS. S/D solutions (20× stock) were added to each sample of virus in PBS or in BSA‐PBS with 10%, 16%, or 25% BSA to achieve the final concentration of TNBP (0.3% v/v), specifically, and either 1% Tween 80, 1% Triton X‐100, or 0.2% sodium cholate. An aliquot was removed from each of the samples and diluted tenfold (Tween 80 and sodium cholate) or 100‐fold (Triton X‐100) in DMEM after 2, 4, 6, and 24 h of incubation at room temperature. The dilution step was necessary to stop the inactivation reaction and to negate the cytotoxic effects of the S/D on the Vero cells during the titration analysis. They observed differences according to the method used for the inactivation of SARS‐CoV‐1. The treatment of SARS‐CoV‐1 with TNBP/Triton X‐100 resulted in the inactivation of the virus below the LOD within 2 h, regardless of the buffer used (PBS or BSA‐PBS solutions), which is in accordance with [65]. By contrast, when TNBP/Tween 80 was used, it took 2 h for the virus to be inactivated in PBS and 10% BSA. However, 4 h were required to inactivate the virus to the LOD in 16% and 25% BSA protein solutions. The treatment of SARS‐CoV‐1 with TNBP/sodium cholate shows greater differences according to the formulation of the buffer (PBS or BSA‐PBS). When using PBS only, 2 h are necessary to inactivate the virus below the LOD, and 4 h are needed in the case of 10% BSA solution. The virus titer in 16% and 25% BSA protein solutions was still detectable after 24 h. These results suggest that SARS‐CoV‐1 inactivation by S/D treatment can be effective, but the incubation time is among the key parameters here.

In addition, in one study, the authors evaluated the effect of the detergent Triton‐X on protein 3a [36]. As mentioned above, the 3a protein is one of the viral proteins that is expressed abundantly in infected cells. This protein is localized in intracellular and plasma membranes and is also found in association with intracellular SARS‐CoV particles. The authors showed that 3a protein expression alone was enough for its release in membrane‐bound structures with buoyant densities of *ρ* = 1.14–1.16 g/mL. Furthermore, membrane flotation analysis indicated that at least some of the 3a proteins in the membrane structures were resistant to detergent treatment, suggesting that a subpopulation of the released 3a protein is associated with detergent‐resistant membranes. Here, 1% Triton‐X at 4°C or room temperature have been employed.

#### 5.2.4 Inactivation procedures to develop killed‐virus vaccines

The proper virus inactivation is of key importance. If inactivation is not complete, viral outbreaks may occur after vaccination. On the other hand, if the virus epitopes are destroyed during inactivation, it may result in a poor neutralizing antibody response and poor protection. The different methods for various types of viruses have been reviewed [66]. In this article, we will focus on the results obtained for the SARS‐CoV viruses.

**Figure 1:**
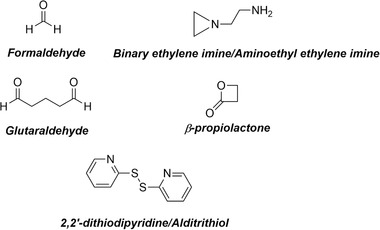
Chemical reagents most commonly used to inactivate viruses for the development of killed‐virus vaccines.

To inactivate the virus, the most commonly used chemical reagents are (Fig. 1): formaldehyde (crosslinker and alkylating agent), glutaraldehyde (crosslinker), 2,2′‐dithiodipyridine/alditrithiol (crosslinker), β‐propiolactone (alkylating agent and crosslinker), and binary ethylene imine/aminoethyl ethylene imine (alkylating agent). A detailed description of the mechanisms and modes of action are given in the Supporting Information. The studies mentioned therein have been mainly conducted on SARS‐CoV‐1. However, these results can probably be extended to SARS‐CoV‐2. Formaldehyde can be applied well, if the right inactivation conditions have been found [52]. The reagent β‐propiolactone was effectively applied for SARS‐CoV‐1 [67, 68], and it has already been successfully employed for a SARS‐CoV‐2 project (see section 7 “Formulation”, [12]). Furthermore, aminoethyl ethylene imine successfully inactivated several noncorona viruses, as given in the Supporting Information.

## 6 Stability during the downstream processes

For vaccine production or virus analysis, downstream processes are necessary, whether it is to produce the vaccine or to produce the virus for further studies. From the already published articles about downstream processes, it is possible to extract some information about the overall virus stability. These protocols are typically not optimized for recovery or stability yet, as normally, the intention is only “good (enough) for purpose”. However, a number of useful laboratory protocols regarding coronaviruses, including sucrose density gradient centrifugations, have been provided [69]. It was also shown that coronavirus preparations can be stable after centrifugation at least 10 000 × *g* at 4°C for 20 min [70].

The full SARS‐CoV‐1 was expressed in the Vero cell line and harvested from the supernatant via 10% PEG precipitation, followed by a sucrose density gradient ultracentrifugation. The virus was suspended in HEPES‐buffered saline (0.15 M NaCl, 20 mM HEPES, pH 6.8) and seemed to be stable under these conditions [23].

A SARS‐CoV‐1 BJ‐01 strain has been cultivated and inactivated in another work, using Vero cells as well. The viruses, concentrated through ultrafiltration, were suspended in PBS and purified via SEC, followed by anion exchange chromatography, and were analysed via HPLC [67].

The SARS‐CoV‐1 E protein was expressed for bioinformatics analysis and in this study at least, the glutathione *S*‐transferase (GST) tag‐fused E protein was able to withstand the following buffer conditions: 50 mM Tris‐HCl, pH 8.0, 1 mM EDTA, 300 mM NaCl, 1 mM DTT, 1 mM PMSF, and sonication in an ice bath for 30 min. Moreover, high centrifugation forces of 14 000  ×  *g* and the buffers for the glutathione S‐transferase (GST) column purification process did not affect it significantly. At last, the protein E was stored in a typical buffer, consisting of 20 mM sodium phosphate and 100 mM NaCl, with a pH of 7.4. Since the authors only intended to speedily analyse the protein E, no information can be derived regarding the long‐term stability [71].

A different approach for vaccine production was investigated by the expression of SARS‐CoV‐1 or MERS‐CoV S proteins. After *Spodoptera frugiperda* cell (Sf9) lysis, the nonsoluble parts were removed via centrifugation 10 000 × *g* for 30 min and the S proteins remained in the supernatant. These proteins formed nanoparticles (about 25 nm in size) among themselves. These nanoparticles were injected into mice for immunization experiments. The SARS‐CoV‐1 group without any adjuvant had the lowest antibody titer, the group with 120 µg aluminium hydroxide had a higher titer (7‐ to 11‐fold), and with 5 µg Matrix M1, the highest neutralizing antibody titer (36‐ to 39‐fold) occurred. Similar results were obtained for the MERS‐CoV S protein with various S protein doses [72].

## 7 Formulation

Developing a vaccine against SARS‐CoV‐1 has been a declared objective of research ever since the outbreak in 2002. Such an outbreak could have occurred anytime again, and thus, the healthcare systems were in dire need of a functional and safe vaccine. A review about SARS‐CoV‐1 vaccines was published in 2009 [73], and in 2012, another review became available, presenting a roadmap for their intended production of a vaccine based on the RBD from the S protein (RBD‐S). This vaccine should be formulated together with alum and a toll‐like receptor 4 (TLR4) agonist like glucopyranosyl lipid A (GLA) as adjuvants, as they are known to enhance the antibody titers. It was argued that the use of RBD‐S only results in fewer side effects, such as eosinophilic and immune enhancing pathologies, which are known to occur when using complete recombinant S proteins or inactivated viruses. The authors assumed that they will be able to finish the development and pretesting of this desired SARS‐CoV‐1 vaccine for adults (older than 15 years) within 5 years and then be able to enter phase I of the clinical trials [74].

Even though this review is an interesting read, data related to this roadmap have not yet been published. Furthermore, much faster vaccine developments are certainly possible for SARS‐CoV‐2 by global collaboration and parallel research. Very recently, two reviews that are well worth reading tried to sum up all the activities and considerations in the past few months [75, 76].

During the SARS‐CoV‐1 outbreak, 23 amino acid changes were noticed in the S protein, and 23 neutralizing mAbs against the S protein could be identified; at least three of them were distinctly mapped. The three most broadly neutralizing mAbs (S109.8, S227.14, and S230.15) were further investigated for their ability to protect against a deadly SARS‐CoV‐1 immunization challenge. It is not understood how S109.8 neutralizes the S protein, but S230.15 seems to block the interaction with the hACE2 receptor. In addition, S227.14 seems to slightly overlap with the recognized epitopes from S230.15. Mice injected 24 h before a mild challenge with 25 µg S227.14 and S230.15 were protected from significant weight loss. This amount was not sufficient when using S109.8, but a tenfold increase of the dose resulted in total protection by all three antibodies. Using the most broadly neutralizing mAb S230.15, different injection times were investigated, whereby the best results were obtained 1 day prior or on the same day as the lethal dose injection [77].

VLPs were also investigated as additional vaccine candidates for SARS‐CoV‐1 [78]. Chimeric SARS‐CoV‐1 VLPs containing the S protein and the influenza matrix protein M1 were able to elicit a high titer of neutralizing antibodies. Moreover, it was shown that a single intranasal‐immunization dose of 0.8 µg VLP without aluminium is sufficient to protect mice from a deadly intranasal challenge with two lethal dose 50 (LD_50_) amounts of mouse‐adapted SARS‐CoV strain v2163 on day 42 [79].

Another study compared a SARS‐CoV‐1 DNA vaccine, which consisted of a plasmid encoding the S protein under the control of the human cytomegalovirus (CMV) promoter and intron A, an inactivated virus, as well as a combination of both. The most effective is the combination of the two, but it elicits a similar immune response as a double injection of the inactivated virus. Furthermore, the combination vaccine induced T‐helper type 1 (Th1) immune responses and the inactivated virus T‐helper type 2 (Th2) immune responses. These combination vaccines seem promising [80].

Immunity against SARS‐CoV‐1 often rapidly wanes. Without adjuvants, much lower titers of neutralizing antibodies are achieved. Different recombinant S proteins and inactivated virus candidates alone, or in combination with alum, cytosine‐phosphate‐guanosine (CpG) oligodeoxynucleotides, or a delta inulin‐based polysaccharide (Advax™) adjuvant were applied to mice models. Preservative‐free Advax‐1™ and Advax‐2™, which contain 10 µg CpG, were also tested. Advax‐1™ enhanced the IgG1 response significantly, which was maintained up to 1 year after immunization. Advax‐2™ enhanced IgG1, IgG2a, IgG2b, and IgG3, and also maintained it for 1 year. CpG enhanced IgG2a, IgG2b, and IgG3, and also maintained it for 1 year. However, the antibody titers were higher with the Advax™ adjuvants. All vaccines were able to protect mice infected with an LD_95_ dose, whereby adjuvant‐free vaccines were less protective. This study also showed, that Advax™ was not only able to induce protection against clinical symptoms, but also reduced the occurrence of lung eosinophilic immunopathology [81].

The importance of the properly chosen adjuvant was emphasized in [67] as well. Monkeys were infected with a vaccine from an inactivated SARS‐CoV‐1 BJ‐01 strain, to investigate the ability to elicit the immune system. Groups were injected with the purified virus and an adjuvant (aluminium hydroxide, aluminium content: β = 0.5 mg/mL), only purified virus, nonpurified virus, and as the control, Vero cell supernatant only. The vaccine with the adjuvant had the highest antibody titer, whereby the titers from the nonadjuvant and nonpurified group were similar. No antibodies were detected in the control group. A virus challenge infection with SARS‐CoV‐1 showed that the vaccinated monkeys developed no symptoms and no SARS‐CoV‐1 genetic information was obtainable from them [67]. Another protocol to produce a SARS‐CoV‐1 vaccine from S protein with a delta‐inulin adjuvant has also been given [82]. Possible suitable adjuvants for SARS‐CoV‐2 vaccines have already been discussed comprehensively [75].

Gao et al. (2020)[12] investigated a simple, but very promising, method for producing a vaccine against SARS‐CoV‐2 by inactivating a SARS‐CoV‐2 strain for 24 h with β‐propiolactone, purifying it with ion exchange and SEC, followed by adding alum as the adjuvant. This study is not yet peer‐reviewed, but it is already available online. Eleven different strains were taken from hospitalized patients worldwide (five from China, three from Italy, one each from Spain, Switzerland, and the United Kingdom). For the development of the inactivated virus vaccine, strain CN2 (China) was chosen, inactivated and combined with alum. Mice were vaccinated with a two‐injection schema (0, 1.5, 3, or 6 µg per dose). The vaccine could elicit higher levels of S‐specific antibodies than found in COVID‐19 patients. Neutralizing antibodies were also detected. This vaccine also elicited neutralizing antibodies against the other collected SARS‐CoV‐2 strains and provided protection. In Wistar rats, similar results could be achieved. Furthermore, because they develop a disease similar to COVID‐19, rhesus macaques were immunized at days 0, 7, and 14 (3 or 6 µg per dose). The antibody titers at week three were similar to the ones from COVID‐19 patients. After this, a SARS‐CoV‐2 CN1 challenge was directly applied into the animals’ lungs. No virus was detectable in the high‐dose vaccination group at day 7 and it was only partially detectable in the medium‐dose group. However, the virus load was around 95% lower than the control group. Interestingly, no antibody‐dependent enhancement was observed. In SARS‐CoV‐1 or MERS‐CoV vaccine development, vaccines caused issues with pulmonary immunopathology. This was further investigated within a second group, but haematological and biochemical analyses did not reveal significant differences to the control groups [12].

## 8 Electrophoresis

There is only little knowledge about the analytical characterization of SARS‐CoV or its proteins. The N protein of SARS‐CoV was expressed in yeast, HEK293 cells, and *Escherichia coli*, and was investigated by two‐dimensional electrophoresis (2DE). Just a single spot appeared using *E. coli*, but multiple spots arose using the eukaryotes, indicating post‐translational modifications in these cell cultures. The two‐dimensional electrophoresis (2DE) spots were also analyzed by Western blotting and MALDI‐TOF/TOF‐MS [83]. Then, an SDS‐PAGE method was used to characterize Nsp2 from COS‐7 cells [84]. However, two excellent reviews describe the possible conditions for the characterization of viruses in general [85, 86]. Based on Kremser et al. [85], methods to characterize rhinovirus subviral A particles and papillomavirus 6b L1 VLPs have been developed, using slightly alkaline borate buffers containing the tenside Thesit® [87, 88]. Furthermore, two following recent articles are highly recommended for an overview of this field, even though they deal with adenoviruses, which are quite different to *Coronaviridae* [89, 90]. For example, a buffer containing 125 mM Tris, 338 mM tricine (pH 7.7), and 0.2% Tween‐20 has been used in a PVA‐coated capillary to determine adenovirus concentrations during vaccine manufacture.

As an educated guess, we presume that electrophoresis can be quite useful to characterize SARS‐CoV‐2 and its proteins. Aggregation must certainly be expected (compare [91]), and coated capillaries may be advisable to reduce adsorption. The rather lipophilic properties of the virus and its proteins may complicate the use of surfactants or organic solvents (see section 5.2.3 “Solvent/Detergent”). Still, we think that the right surfactants in a reasonable concentration may be useful for *Coronaviridae* as well. The information given in section 2 “Size and structure” and Table 1 can support the understanding of the obtained separations. In general, patterns obtained by CIEF or CZE can possibly very well characterize the purity and the stability of a viral product, for example, a killed‐virus vaccine.

## 9 Quality attributes of vaccines

The quality attributes of vaccines are best defined in the European Pharmacopoeia (Vaccina at usum humanum) [92] and the United States Pharmacopoeia (USP, 〈1235〉) [93]. Numerous individual vaccines are also described in these pharmacopoeiae, demonstrating that vaccines are very diverse. The same applies to their quality attributes. However, tests for identity, content uniformity, potency, and safety and product‐related impurities are always essential. Further, stability tests are vital: one can find additional guidance about this topic in the ICH guideline Q6B [94] and the USP monograph 〈1049〉 [95].

Identity confirmation, content uniformity, and the evaluation of product‐related impurities and stability can be supported by approaches discussed in sections 6, 7, and 8. The information from sections 2 to 4 may help to evaluate the obtained analytical data. Potency, and possibly safety, investigations can be supported by the assays described in sections 5, 6, and 7.

## 10 Concluding remarks

It is impressive to see how fast the research of SARS‐CoV‐2 and the development of a vaccine is progressing, even though parts of the picture are certainly missing after half a year of intensive global research.

SARS‐CoV‐1 and SARS‐CoV‐2 are quite similar in many regards, having a sequence similarity of approximately 75% and very similar (predicted) p*I* values of the respective corresponding proteins. Therefore, information can often be derived for the new virus. For example, disinfection efficacies have been successfully obtained from SARS‐CoV‐1 experiences. However, there are also important differences.

The structural proteins S, E, and M could be of utmost importance for the development of potential vaccines, as they play a key role in the entry of the virus into the host cell or the assembly of the virus. We have elaborated that the S proteins of both variants are seemingly very similar, but apparently small differences have strong effects, for example, on the epitope setting. Unevaluated knowledge transfer from SARS‐CoV‐1 to SARS‐CoV‐2 can thus not be advised, but it is possible to use SARS‐CoV‐1 data as a reference and a starting point for investigations.

Both SARS‐CoV‐1 and ‐2 are unusually stable, for example, on surfaces, but as enveloped viruses, they are rather lipophilic and therefore sensitive to solvents and surfactants. Various typical disinfectants are effective, such as those containing ethanol or 2‐propanol. The virus is also sensitive to surfactants, as typical for enveloped viruses. However, an unusual pH stability has been observed. Different methods of virus inactivation have been evaluated. The most important parameters are the incubation time and temperature. The agent ß‐propiolactone is possibly a good choice for inactivating viruses with regard to killed‐virus vaccines.

The stability of the whole virus or SARS VLPs during the downstream process is not yet comprehensively covered in the literature evaluated here. Only limited data can be found regarding formulation. Strategies for vaccine product qualities still need to be defined, but quality assurance methods can be derived from the existing methods related to other investigations on SARS‐CoV‐2, as we have outlined in this work.

Certainly, further investigation needs to be done in these areas. However, several pieces of information, which show that standard approaches employed for other viruses and vaccines seem to be feasible, including centrifugation conditions and the use of adjuvants, are given.

*We are grateful to Prof. Dr. Ingo Ott for critically reading an earlier version of the manuscript*.

*The authors have declared no conflict of interest*.

## Supporting information

Supporting Information.
Mechanisms of action of different chemical reagents most commonly used inactivation procedures for viruses for development of killed‐virus vaccines.
