# Supplementary material for: Physicochemical properties of SARS‐CoV‐2 for drug targeting, virus inactivation and attenuation, vaccine formulation and quality control
Source: Electrophoresis. 2020 Jun 8;41(13-14):1137–51. doi: 10.1002/elps.202000121 (PMC7283733; doi:10.1002/elps.202000121)
Supplement: Supplementary file 1 — Supporting Information. Mechanisms of action of different chemical reagents most commonly used inactivation procedures for viruses for development of killed‐virus vaccines. [file ELPS-41-1137-s001.docx]

Physicochemical properties of the SARS-CoV-2 for drug targeting, virus inactivation and attenuation, vaccine formulation and quality control

C. Scheller^1^, F. Krebs^1^_,_ R. Minkner^1^_,_ I. Astner^2^_,_ M. Gil Moles^1^_,_ H. Wätzig^1,^*

Table of contents:

Mechanisms of action of different chemical reagents most commonly used inactivation procedures for viruses for development of killed virus vaccines.

# Formaldehyde (crosslinker and alkylating agent)

Based on its electrondeficient carbon atom, formaldehyde has the ability to interact with the genome and proteins. The non-protonated exocyclic amine on adenine (N6) or non-protonated amino groups of the N-terminal amino acids (lysine, arginine, glutamine, tryptophan and histidine) and sulfhydryl groups, such as present in cysteine, can act as a nucleophile and react with the electrophilic part of formaldehyde. Initially a monohydroxymethylation (N6 adenine DNA or RNA and N-terminal amino acids) takes place, as shown in **Figure S1**. Subsequently, a water molecule can be released resulting in a Schiff base formation. The imine intermediate can then crosslink by a nucleophilic reaction. These crosslinking reactions can also occur between genome and proteins.





**Figure S1**: Mechanism of the interaction of formaldehyde with DNA/RNA

Some formaldehyde inactivated vaccines work fine, however, in other cases problems can be observed. For example, formaldehyde inactivation can contain incompletely inactivated virus particles that can cause outbreaks of virus infections. In other cases, the formaldehyde can destroy the viral structure resulting in a poor immune response. However, in the case of the SARS-CoV virus Darnell et al. [1] examined formaldehyde inactivation at different temperatures finding a dependence of the inactivation with the temperature. Also, they studied different concentrations of formaldehyde. Final concentrations of formaldehyde were 0.037% (1:1000) and 0.009% (1:4000) and the virus and aldehyde samples were incubated at 4, 25 and 37 °C for up to three days.

They observed that a concentration of 0.009% (1:4000) and 4 °C the formaldehyde was not able to inactivate the virus even after exposure for three days. In contrast, at 25 and 37 °C formaldehyde inactivated most of the virus, close to the limit of detection of the assay, after one day. However, they did not observe a complete inactivation after three days. On the contrary, when the concentration is 0.037% (1:1000), the inactivation of the virus was complete over the whole range of temperatures and days. However, they could not establish comparisons since the viral load was below the detection limit. These results suggest that inactivation of SARS-CoV with formaldehyde may be a good method, provided that the right conditions are applied.

# Glutaraldehyde (crosslinker)

Glutaraldehyde has similar chemical groups to formaldehyde and therefore the mechanism of action is similar. The glutaraldehyde consists of two aldehydes connected by three saturated carbons. The interaction with the genome (DNA, RNA) of glutaraldehyde is the same as for formaldehyde. In the case of proteins, the mechanism is different; glutaraldehyde forms unsaturated aldehydes by aldol condensation and water elimination. Later some amino acids can be linked to glutaraldehyde via Michael type addition. In this case the intermediate Schiff bases are not formed (see **Figure S2**). These types of reactions can also cause crosslinking of the genome and proteins.





**Figure S2**: Mechanism of the interaction of glutaraldehyde with proteins

The use of glutaraldehyde for virus inactivation may cause some problems. Glutaraldehyde has two electrophilic carbons and after aldolic condensation it becomes a multifunctional crosslinking agent, which can generate extensive damage to the virus. This fact could cause problems with a poor immune response.

In the case of SARS-CoV, Darnell et al. [1] studied glutaraldehyde inactivation. They carried out the study at different concentrations (0.008% (1:1000) and 0.002% (1:4000)) and temperatures (4, 25 and 37 °C) such as for formaldehyde. As in the previous case, when the concentration was 0.008% (dilution factor 1:1000), they could neither determine the effect of the temperature nor of the incubation period (maximum three days) since the viral load was near or below the detection limit. Nevertheless, when the dilution factor is 1:4000 there are differences. At a temperature of 4 °C the virus is not completely inactivated even after three days. On the other hand, when the temperature increases to 25 °C after 2 days of incubation, the virus is completely inactivated and only one day is necessary at 37 °C. These results could be indicating that glutaraldehyde can inactivate viruses, however, their high reactivity can destroy viral proteins causing low protection in same cases.

# β-propiolactone (crosslinker and alkylating agent)

β-propiolactone has been widely used for virus inactivation with vaccine applications. The main mechanism by which β-propiolactone acts is through the alkylation of guanine present in viral DNA or RNA. But it is important to note that β-propiolactone can also interact with proteins through an acylation reaction. In **Figure S3** the difference between alkylation and acylation is shown.





**Figure S3**: Difference between alkylation and acylation.

The mechanism by which β-propiolactone reacts with the genome is through an alkylating reaction between the N7-guanine or N7-adenine (nucleophile) and the electrophilic carbon of the β-propiolactone. This nucleophilic substitution causing the ring opening of β-propiolactone and the N-alkylation of nucleotide is shown in **Figure S4**.





**Figure S4**: Mechanism of the reaction of β-propiolactone with DNA/RNA

In contrast, the reaction of β-propiolactone with proteins occurs through an acylation mechanism between the carbonyl present in β-propiolactone and the nucleophile part of amino acids. The most reactive residues are cysteine, methionine and histidine and, to a lesser degree, aspartic acid, glutamic acid, tyrosine, lysine, serine, and threonine (see **Figure S5**) [2].





***Figure S5****: Mechanism for the interaction of β-propiolactone with proteins.*

The inactivation of viruses is typically performed with β-propiolactone concentrations between 0.025 and 1%. For the SARS-CoV there are several studies, in which β-propiolactone has been used for the inactivation of SARS-CoV where the production of antibodies in animal models has been evaluated.

To observe whether the SARS inactivated vaccines induced SARS-specific neutralizing antibodies, Cynomolo-gus macaques were immunized with adjuvant-containing purified vaccine, purified vaccine and unpurified vaccine. In this case for inactivation of the virus β-propiolactone was used and the following method was employed. β-propiolactone was added at 1:4000 (v/v) dilution. The shaken mixture was kept at 2–8 ◦C for more than 16 h before incubating at 37 ◦C for 2 h to inactivate the virus completely. In this study they observed that the purified inactivated SARS vaccine induces a high production of neutralizing antibody and protects the monkeys after a SARS-CoV challenge [3].

Another study was conducted using mice and hamsters as animal models. In this case the authors used a different method for virus inactivation. They used β-propiolactone at 1:1000 (v/v) dilution and incubated for 24 h at 48 °C. This was followed by a second incubation for 24 h at room temperature to hydrolyze residual β-propiolactone. The results obtained in this study reveal that the non-adjuvanted SARS vaccine was immunogenic and provided a high level of protection from early challenge in mice and hamsters. However, more robust protection from late challenge was observed in animals immunized with adjuvanted SARS vaccine than in animals immunized with the non-adjuvanted WI-SARS vaccine [4].

In conclusion, β-propiolactone could be a good candidate for inactivation of SARS virus by chemical methods.

# Aminoethyl ethylene imine (alkylating agent)

Aminoethyl ethylene imine has two protonizable amino groups and has a high affinity to the genome. At low concentration the aminoethyl ethylene imine can pass through the virus capsid and only alkylates the genome. The mechanism of action is like the one of β-propiolactone. N7-guanine or N7-adenine (nucleophile) reacts with the electrophilic carbon of aminoethyl ethylene imine causing opening aminoethyl ethylene imine ring and N-alkylation of the genome (see **Figure S6**). For the moment there are no studies on the interaction of aminoethyl ethylene imine with proteins, probably because it is usually used at low concentrations. This suggests that the viral epitopes in surface of the virion are preserved after treatment of the virus with the aminoethyl ethylene imine. Most vaccines based on aminoethyl ethylene imine inactivated virus are able to induce a protective immune response. There are examples for existing studies on many viruses, such as FMDV [5], RRV [6], sheep pox [7], HIV [8], Nipah virus [9], HNV [10] and PRRSV [11]. However, no studies have yet been conducted on the SARS-CoV virus.





**Figure S6**: Mechanism of the reaction of Aminoethyl ethylene imine with DNA/RNA

# Aldrithiol or 2,2’-dithiodipyridine (crosslinker)

Aldrithiol has the ability to interact with proteins, in particular aldrithiol oxidizes the sulfhydryl groups present in cysteine residues, which results in formation of S-S bridges that crosslink proteins (see **Figure S7**). Nevertheless, it should be noted that intracellular and extracellular cysteine residues are exposed to different environments. The internal viral proteins are in a reducing environment, and cysteine is in the reduced thiol form. In contrast, the surface proteins of viruses are subjected to an oxidizing environment, the cysteine are present as disulfides (S-S). Probably only the viruses where free thiols are important for the virus infection can be inactivated by aldrithiol.





***Figure S7****: Mechanism of the interaction of Aldrithiol with proteins*

Some studies have been conducted on the inactivation of viruses with aldrithiol. For example, aldrithiol can inactivate HIV [12] and SIV [13] properly, but vaccination studies could not determine if aldrithiol-inactivated virus can protect the host on challenge. As in the previous case (aminoethyl ethylene imine), there are no studies on the SARS-CoV yet.

References

[1] Darnell, M. E. R., Subbarao, K., Feinstone, S. M., Taylor, D. R., *Journal of Virological Methods*. 2004, *121* (1), 85–91, 10.1016/j.jviromet.2004.06.006.

[2] Uittenbogaard, J. P., Zomer, B., Hoogerhout, P., Metz, B., *The Journal of biological chemistry*. 2011, *286* (42), 36198–36214, 10.1074/jbc.M111.279232.

[3] Qin, E., Shi, H., Tang, L., Wang, C., Chang, G., Ding, Z., Zhao, K., Wang, J., Chen, Z., Yu, M., Si, B., Liu, J., Wu, D., Cheng, X., Yang, B., Peng, W., Meng, Q., Liu, B., Han, W., Yin, X., Duan, H., Zhan, D., Tian, L., Li, S., Wu, J., Tan, G., Li, Y., Li, Y., Liu, Y., Liu, H., Lv, F., Zhang, Y., Kong, X., Fan, B., Jiang, T., Xu, S., Wang, X., Li, C., Wu, X., Deng, Y., Zhao, M., Zhu, Q., *Vaccine*. 2006, *24* (7), 1028–1034, 10.1016/j.vaccine.2005.06.038.

[4] Roberts, A., Lamirande, E. W., Vogel, L., Baras, B., Goossens, G., Knott, I., Chen, J., Ward, J. M., Vassilev, V., Subbarao, K., *Viral immunology*. 2010, *23* (5), 509–519, 10.1089/vim.2010.0028.

[5] Bahnemann, H. G., *Archives of Virology*. 1975, *47* (1), 47–56, 10.1007/BF01315592.

[6] Aaskov, J., Williams, L., Yu, S., *Vaccine*. 1997, *15* (12-13), 1396–1404, 10.1016/S0264-410X(97)00051-0.

[7] Awad, M., Michael, A., Soliman, S. M., Samir, S. S., Daoud, A. M., *The Egyptian journal of immunology*. 2003, *10* (2), 67–72.

[8] Race, E., Frezza, P., Stephens, D.M., Davis, D., Polyanskaya, N., Cranage, M., Oxford, J. S., *Vaccine*. 1995, *13* (1), 54–60, 10.1016/0264-410x(95)80011-2.

[9] Berhane, Y., Berry, J. D., Ranadheera, C., Marszal, P., Nicolas, B., Yuan, X., Czub, M., Weingartl, H., *Journal of Virological Methods*. 2006, *132* (1-2), 59–68, 10.1016/j.jviromet.2005.09.005.

[10] Anderson, E., Clouthier, S., Shewmaker, W., Weighall, A., LaPatra, S., *Journal of fish diseases*. 2008, *31* (10), 729–745, 10.1111/j.1365-2761.2008.00960.x.

[11] Vanhee, M., Delputte, P. L., Delrue, I., Geldhof, M. F., Nauwynck, H. J., *Veterinary research*. 2009, *40* (6), 63, 10.1051/vetres/2009046.

[12] Rossio, J. L., Esser, M. T., Suryanarayana, K., Schneider, D. K., Bess, J. W., Vasquez, G. M., Wiltrout, T. A., Chertova, E., Grimes, M. K., Sattentau, Q., Arthur, L. O., Henderson, L. E., Lifson, J. D., *Journal of virology*. 1998, *72* (10), 7992–8001.

[13] Lifson, J. D., Rossio, J. L., Piatak, M., Bess, J., Chertova, E., Schneider, D. K., Coalter, V. J., Poore, B., Kiser, R. F., Imming, R. J., Scarzello, A. J., Henderson, L. E., Alvord, W. G., Hirsch, V. M., Benveniste, R. E., Arthur, L. O., *AIDS research and human retroviruses*. 2004, *20* (7), 772–787, 10.1089/0889222041524661.
